# When, where, and why specialised metabolites are produced: inferring function from expression control

**DOI:** 10.1042/EBC20250024

**Published:** 2026-08-19

**Authors:** Sébastien Rigali

**Affiliations:** InBioS—Center for Protein Engineering, University of Liège, Institut de Chimie B6a, Liège B-4000, Belgium

**Keywords:** antibiotics, biosynthetic gene cluster, cryptic metabolites, gene regulatory networks, Microbial ecology, Natural product discovery

## Abstract

Although some microbial compounds have been repurposed for human use, microorganisms did not evolve their specialised metabolites with us in mind. Many natural products likely possess hidden activities, while others may be exploited in ways that ignore their most biologically relevant roles. Uncovering the true function of these compounds is essential not only for understanding microbial interactions in native environments but also for unlocking their most appropriate use. To facilitate prioritisation in discovering new natural products, computational tools have been developed to predict the function of compounds hidden in cryptic biosynthetic gene clusters. Yet beyond *in silico* predictions, understanding when, where, and why metabolites are produced is critical for both fundamental biology and targeted discovery. After all, what nature chooses to activate at a specific time or condition tells us what it is really for. Based on the principle ‘function follows regulation’, it is no coincidence that expression of metal chelators, phytotoxins, pigments, and antibiotics is controlled by metal availability, plant byproducts, radiations, and competitor sensing, respectively. Likewise, metabolite localisation and production timing also provide clues to function such as intracellular antiproliferative agents coordinating programmed cell death or pigments protecting against oxidative stress. These controlled expression patterns suggest a strategic approach for natural product discovery: focusing on culture conditions that mimic the environmental or developmental contexts under which metabolites are needed for the producer. Integrating expression control information offers a predictive framework to guide experimental design, increases the likelihood of identifying compounds with meaningful ecological roles, and anticipates their applications.

## Introduction

Biosynthetic gene clusters (BGCs) are ubiquitous across microbial genomes, driving the production of an extraordinary diversity of specialised metabolites. The shift in terminology from ‘secondary’ to ‘specialised’ metabolites reflects a growing recognition that their ecological functions in nature are likely fundamental rather than accessory [1–6]. Yet, the true roles of many bioactive metabolites in the native habitats of their producers remain poorly understood. One of the most debated examples concerns antibiotics: do they function solely as antimicrobials, or do they also play broader ecological and signalling roles [7–12]? The uncertainty surrounding biological function is even greater for metabolites that humans exploit for anticancer, immunosuppressive, or cholesterol-lowering purposes. While microorganisms synthesise antibiotics because they too require antimicrobial defence mechanisms, evolution did not shape their specialised metabolites to treat cancer, prevent organ rejection, or lower cardiovascular risk. As evolution did not ‘design’ tacrolimus to prevent transplant rejection nor ferroverdins to lower cholesterol, the question naturally arises: what functions do these molecules fulfil for their microbial producers?

Unraveling the true functions of microbial metabolites is primarily necessary to better understand how microorganisms interact and communicate in their native habitats. This pursuit alone provides a convincing and exciting rationale for in-depth fundamental research. At the same time, accurately identifying the natural functions and targets of these metabolites will ultimately allow us to align them with the most suitable applications in medicine, agriculture, and biotechnology and to anticipate side effects. Most, if not all, microbial metabolites have never undergone comprehensive bioactivity screening. Large-scale testing platforms remain scarce, and many of the thousands of known microbial compounds were discovered in academic laboratories, where screening typically targets only a few biological activities. Once a short-term project is discontinued, the exploration of the possible applications of a new compound often ends as well. Industrial partners, meanwhile, are hesitant to pursue investigations on promising leads from academic laboratories, data on cytotoxicity being absent and/or fearing poor returns on investment lacking secure intellectual property. Consequently, countless metabolites, including potential hidden gems, remain unexplored, leaving us with a fragmented view of their true activities and possible uses.

The discovery of thousands of cryptic BGCs has amplified interest in natural product research but also underscored the need for prioritisation [13–16]. Neither academic nor industrial programmes can afford to spend years activating clusters only to obtain products irrelevant to their initial objectives. A consortium seeking plant growth-promoting compounds will not celebrate the discovery of another antibiotic. Likewise, pharmaceutical companies are unlikely to pursue molecules with insecticidal activity if their focus is on human therapeutics. While the unpredictability of cryptic BGCs is intellectually appealing for researchers, as it promises novel chemistry and new modes of gene regulation, it is far less convincing to investors. Hardly any are persuaded by the notion of treating a cryptic BGC as a ‘surprise package’ since the mere prospect of discovering something new rarely justifies substantial investment. Stakeholders want to know what they are likely to find and why it matters. To mitigate this uncertainty, computational tools have emerged to predict the structures and functions of metabolites encoded by cryptic BGCs. These algorithms are steadily improving in accuracy, offering varying levels of reliability depending on the genetic information available within each cluster. Such predictive approaches are becoming indispensable for de-risking discovery, guiding investment, and unlocking the full potential of microbial genomes [16].

## From expression regulation to function and target

**Figure 1:**
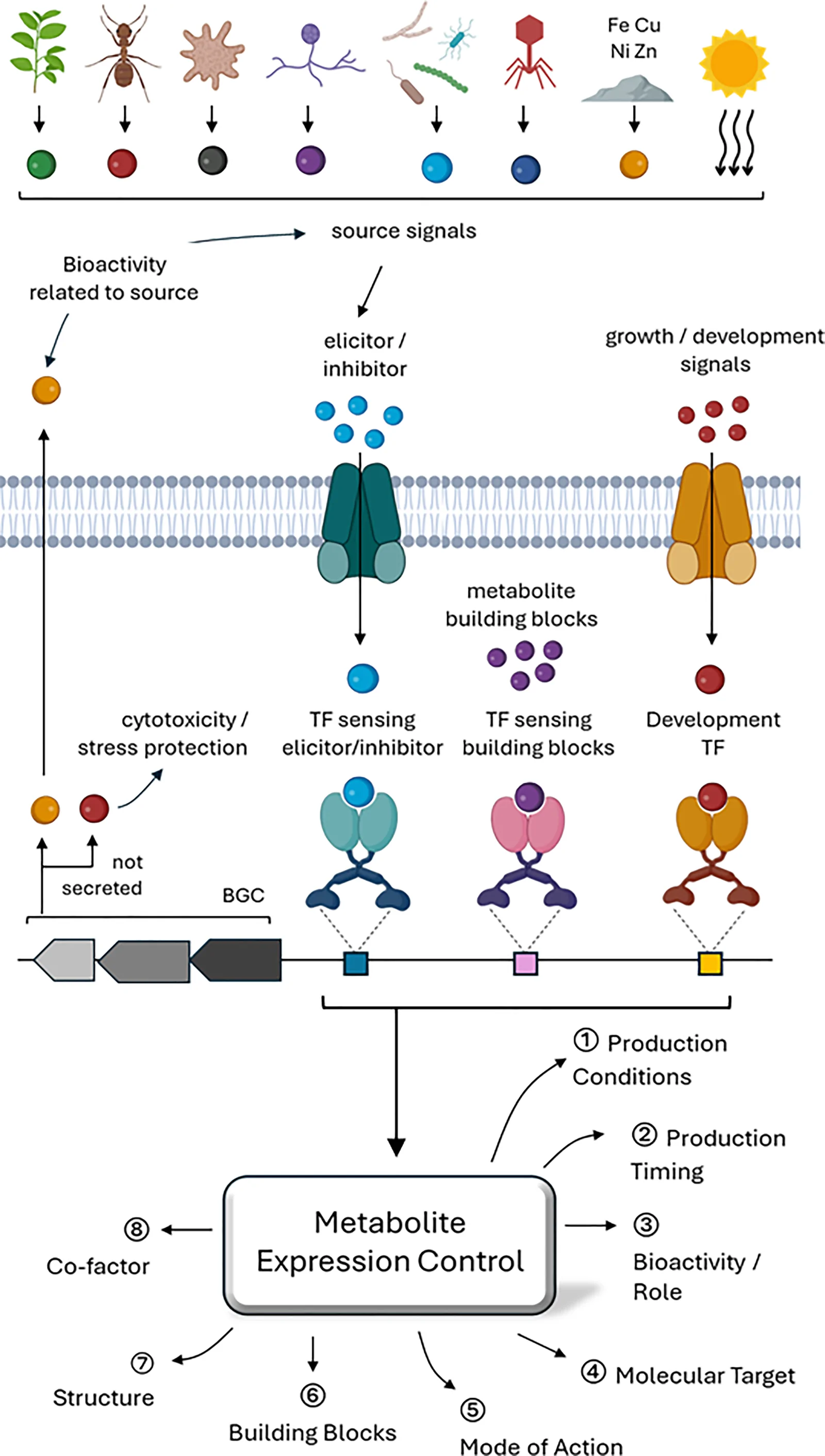
Functional features of natural products potentially inferred from BGC expression control Regulatory pathways link environmental signals to the synthesis of signal-associated bioactive compounds. Insights that can be derived from the types of transcription factors regulating BGC expression include the conditions required for metabolite production (1), developmental timing of expression (2), biological role and function of the natural product (3), molecular targets (4), mode of action (5), necessary precursors for synthesis (6), structural characteristics (7), and cofactors required for activity and structural integrity (8). Where a metabolite accumulates can also suggest its function: intracellular cytotoxic compounds may participate in programmed cell death to coordinate bacterial development, while intracellular pigments may serve to protect against oxidative stress. Created with BioRender.com.

One crucial yet always overlooked factor in predicting the function of a microbial metabolite is the regulation of its expression [17]. Studies on the transcriptional regulation of BGCs are usually motivated by the goal of enhancing the expression of cryptic clusters, thereby facilitating the discovery of their associated compounds [17]. From a broader perspective, investigating regulatory networks highlights links between environmental conditions and the response of bacterial specialised metabolism. Understanding how BGCs are globally and specifically controlled, the environmental cues that activate or repress them and the timing of their expression during growth or differentiation can also yield valuable insights into the ecological roles of their metabolites (Figure 1). In other words, what nature chooses to activate at a specific time or condition tells us what it is really for. After millions of years of evolution, it would be naive to assume that BGC regulatory networks are anything but finely tuned to the specific needs of the producing organism. Given the rapid adaptability of microorganisms to changing environments, uncontrolled or nonspecific BGC expression is highly unexpected. These systems ensure that metabolites are synthesised only when beneficial, in response to precise physiological or environmental conditions and with a well-defined purpose. To effectively address the question, ‘what is hidden within a cryptic BGC?’, researchers in drug discovery must also ask when, where, and why the producer requires the compound encoded within that genetic material [14,15].

A well-established example of this tight regulation–function relationship is siderophore biosynthesis, which is induced under iron-limiting conditions. Here, derepression by Fur-like transcription factors ensures that siderophores are produced only when iron acquisition becomes critical for essential biological processes including host colonisation [18]. This clear regulatory logic suggests a powerful strategy for function prediction: scanning cryptic BGCs for Fur-binding sites should highlight new siderophores with potential applications ranging from conjugates for Trojan horse antibiotics to iron chelation therapies. The same rationale could be extended to the search for chelators of other metals by identifying BGCs possessing binding sites of the regulators of copper, zinc, or nickel homeostasis, among others [19]. However, even in this seemingly straightforward case, regulation is more nuanced than a simple ‘metal off, metal-chelators on’ model. Competition for iron may indeed trigger a more aggressive response such as the release of antimicrobials as a form of counterattack against rivals monopolising vital micronutrients [20–22]. The regulation of non-siderophore metabolites by iron also highlighted another functional link whereby the chelated metal ion serves as a cofactor essential for the metabolite’s structure and activity. A notable example is provided by ferroverdins, where iron not only induces the production of precursor molecules but also coordinates their assembly into the final structure [23]. This coupling ensures that metal-dependent metabolites are produced only when their cofactor is available, optimising both biosynthesis efficiency and ecological fitness.

If the link between expression control and metabolite function is clear for metal chelators, why should it be different for other types of natural products? Thaxtomin phytotoxins produced by *Streptomyces* species that colonise root and tuber crops [24,25] provide another instructive example. Their function is tightly linked to plant cell wall disruption, inhibiting cellulose biosynthesis by interfering with the enzymes that assemble cellulose microfibrils, which ultimately weakens the plant cell wall structure [26,27]. It is therefore no coincidence that the main inducers of thaxtomin production are cellobiose and cellotriose, the very products of cellulose breakdown [28,29]. The bacterium has even repurposed the regulator of its cellulolytic system (CebR), normally devoted to primary metabolism, as the master transcription factor controlling the expression of the thaxtomin BGC [30,31]. This regulatory-functional link is so conserved that CebR-binding sites are maintained within thaxtomin BGCs across almost all *Streptomyce*s species that colonise potato tubers [32]. An interesting exception is found in *Streptomyces ipomoeae*, which colonises sweet potatoes. In this species, thaxtomin expression is not induced by cello-oligosaccharides, and the organism predominantly produces thaxtomin C rather than thaxtomin A [33]. This case highlights how adaptation to a new plant host can rewire biosynthetic regulation and product chemistry, implying that thaxtomin C may act through a distinct mechanism and/or release other signal molecules in sweet potato compared with thaxtomin A in potato. Additionally, regulation of the thaxtomin BGC by Lrp and Lrp2, transcription factors that use phenylalanine and tryptophan as co-activator and co-repressor, respectively, highlights another layer of regulation-structure coupling [34,35]. Because both phenylalanine and tryptophan serve as structural precursors of thaxtomins [36], Lrp- and Lrp2-mediated controls directly link building block availability to specialised metabolite biosynthesis [37]. Consequently, identifying the binding sites of such transcription factors within a BGC can also provide valuable insights into the biosynthetic building blocks of the natural product, offering predictive clues about its composition and structural features.

Another example of tight coupling between regulation and ecological function is provided by allosamidin, a pseudo-trisaccharide that specifically inhibits family 18 chitinases [38]. The regulation of allosamidin biosynthesis and chitinase gene expression are co-induced by chitin or its byproducts [39,40]. Remarkably, allosamidin itself acts as a signalling molecule, stimulating chitinase expression in its producer and creating a positive feedback loop that coordinates the production of the inhibitor and the enzyme it targets [38]. This dual role as both production elicitor and enzyme inhibitor enables fine-tuned control of chitin degradation, preventing excessive enzymatic activity while ensuring efficient nutrient acquisition. This case highlights that stimulating the expression of a specific enzymatic system may, paradoxically, be an effective strategy for discovering inhibitors of the very enzymes whose expression has been induced. A related instance of such regulatory-ecological interplay has also been observed with *N*-acetyl-d-glucosamine (GlcNAc), the monomeric unit of chitin that is a key structural component of both bacterial and fungal cell walls. GlcNAc and other cell wall components serve as potent inducers of antimicrobial production [41–44]. Perception of such molecules may act as a signal of microbial proximity interpreted as the presence of fungal or bacterial competitors and trigger the synthesis of antibacterial and antifungal compounds [41,42,45]. The produced antibiotics themselves can in turn act as signalling molecules inducing specialised metabolite production, including other antibiotics [46]. In these cases, antibiotics, traditionally viewed as offensive/defensive weapons, also function as a signal of environmental stress or microbial competition, resulting in the production of other antibiotics as a self-protective response. Together, these cases exemplify how small molecules derived from structural biopolymers, such as cellulose, chitin, and peptidoglycan, can serve as both metabolic cues and ecological signals, linking nutrient sensing to specialised adaptive responses.

## From expression localisation and timing to function

The localisation of specialised metabolites, whether they are retained within the cell or secreted into the environment, and the timing of their production (early, exponential, and late growth) may also provide important clues about their bioactivity and mode of action. Metabolites that remain intracellular could primarily affect internal cellular processes of the producer, whereas those exported into the environment might act on neighbouring organisms or influence the surrounding environment. Likewise, metabolites produced at specific growth stages are likely to reflect the physiological needs and developmental priorities of the producer at those times. A striking illustration of spatiotemporal control linked to function is the biosynthesis of prodiginines in *Streptomyces coelicolor* [47]. These highly cytotoxic, DNA- and membrane-damaging metabolites [48–50] are retained intracellularly, and their production coincides with a major round of programmed cell death (PCD) during the transition from vegetative growth to sporulation [47]. This PCD event is thought to release nutrients and signal molecules from dying hyphae, supporting the survival and differentiation of neighbouring filaments into spore chains [47,51–53]. Inhibition of prodiginine biosynthesis drastically reduces filament death at this critical stage, leading to premature sporulation [53]. Prodiginines thus act as antiproliferative agents that regulate the balance between survival and sacrifice among filaments, ensuring proper development and delaying the onset of dormancy. Extending the active phase of growth through controlled PCD can be viewed as a microbial strategy to prolong the organism’s productive lifetime. This mechanism conceptually parallels human chemotherapy, where antiproliferative compounds induce selective cell death to sustain overall tissue integrity and function. This analogy suggests that the search for molecules with antiproliferative properties could focus on culture conditions in which bacteria undergo PCD to control cell proliferation during development.

In contrast, several other bacterial pigments retained intracellularly play key roles in protecting the producer from environmental and physiological stress [54,55]. Carotenoids, such as β-carotene, are widespread in radiation-resistant bacteria, where they quench reactive oxygen species and prevent photooxidative membrane damage [56]. Melanins act as powerful antioxidants, shielding cells from UV and oxidative stress and enhancing desiccation tolerance [57,58]. Violacein, produced by *Chromobacterium* and *Janthinobacterium* species, provides protection against oxidative and UV stress through its radical-scavenging properties [59]. In cyanobacteria, scytonemin, localised in the cell wall sheath, screens harmful UV radiation and prevents photooxidative damage [60]. Together, these pigments illustrate a common adaptive strategy among bacteria: the intracellular synthesis of redox-active or UV-absorbing compounds, which stabilise cellular components, modulate internal redox balance, and extend survival under stress. Therefore, the search for new protective pigments for pharmaceutical and cosmetic applications should prioritise intracellular metabolites produced under stress conditions or at critical developmental stages, where their protective functions are most likely to be needed.

## Future directions and conclusions

While inferring the function of microbial specialised metabolites often begins with cataloguing and analysing biosynthetic genes, it is also essential to decode the logic that governs their expression, localisation, and ecological roles. These regulatory frameworks offer crucial insights into a metabolite’s function, inducing conditions, required cofactors, biosynthetic building blocks, structural features, and bioactivity. It is no coincidence that the production of metal chelators, phytotoxins, enzyme inhibitors, protective pigments, or cytotoxic compounds is controlled by cues such as metal availability, plant-derived saccharides, enzyme inducers, UV-light radiation, or cellular stress and death signals, respectively. Regulation is shaped by evolution, such that the environmental signals and transcriptional circuits that activate BGCs generally reflect the natural functions of their products and the needs of the producers. Likewise, factors controlling the timing (early, exponential, or late growth) and spatial localisation (intracellular or secreted, differentiating hyphae) of metabolite production provide essential clues as to whether a compound interferes with intracellular processes, ecological interactions, or developmental transitions.

Integrating regulatory genomics, spatial metabolomics, and ecological context thus offers a powerful framework to predict not only expression conditions of a metabolite but also why it exists and when it matters to the producer. Undeniably, BGC expression is often governed by intricate networks responding to multiple environmental signals, forming a complex puzzle whose pieces must be assembled to reveal the full regulatory and functional picture [61,62]. It will not always be as straightforward as adding plant-derived nutrients and observing the emergence of plant-biology-associated metabolites. But if a metabolite that happens to show antiviral activity is regulated by a transcription factor responsive to plant-derived molecules, it is reasonable to suspect that its true ecological role—and therefore its most relevant application—relates more to plant metabolism/interactions than to antiviral defence against human viruses. Regulation is not an afterthought; it is an evolutionary signature that points directly to the ecological function of a metabolite. Ignoring this dimension in function prediction is not merely incomplete; it represents a fundamental blind spot in our understanding of natural product bioactivities.

If the scientific community has widely accepted the importance of understanding BGC regulation, a major challenge remains. Despite remarkable advances in genomics, most bacterial transcription factors remain uncharacterised, leaving a substantial knowledge gap regarding their DNA-binding sites, allosteric effectors, and the biological processes they control. Bridging this gap is essential to fully understand how environmental cues translate into BGC activation. Emerging computational tools such as COMMBAT [63] will help address this challenge by linking environmental signals to BGC expression, moving the field from serendipitous screening to more rational, hypothesis-driven discovery.

## Summary

- **Why function prediction matters:** Understanding what specialised metabolites do in nature helps explain microbial ecology and guides their most relevant applications.
- **Function follows regulation:** Metabolite production is tightly controlled; when, where, and why a specialised metabolite is expressed reveals its biological purpose.
- **From ecology to discovery:** Decoding expression control transforms natural product discovery from random screening to a rational, prediction-driven process.

## Open Access

Open access for this article is provided via our Subscribe to Open programme, made possible through the support of our subscribing institutions. Learn more here: https://portlandpress.com/pages/open_access_options_and_prices#conditional.

## Abbreviations

BGCs: biosynthetic gene clusters
GlcNAc: *N*-acetyl-d-glucosamine
PCD: programmed cell death

## References

[1] Davies J. (2013) Specialized microbial metabolites: functions and origins. J. Antibiot. (Tokyo) 66, 361–364 10.1038/ja.2013.61 23756686

[2] Schmidt R., Ulanova D., Wick L.Y., Bode H.B. and Garbeva P. (2019) Microbe-driven chemical ecology: past, present and future. ISME J. 13, 2656–2663 10.1038/s41396-019-0469-x 31289346 PMC6794290

[3] Erb M. and Kliebenstein D.J. (2020) Plant secondary metabolites as defenses, regulators, and primary metabolites: the blurred functional trichotomy. Plant Physiol. 184, 39–52 10.1104/pp.20.00433 32636341 PMC7479915

[4] Li J., Baldwin I.T. and Li D. (2022) Harmonizing biosynthesis with post-ingestive modifications to understand the ecological functions of plant natural products. Nat. Prod. Rep. 39, 1383–1392 10.1039/D2NP00019A 35575224 PMC9298679

[5] Zhang Y., Gallant É., Park J.D. and Seyedsayamdost M.R. (2022) The small-molecule language of dynamic microbial interactions. Annu. Rev. Microbiol. 76, 641–660 10.1146/annurev-micro-042722-091052 35679616 PMC10171915

[6] Nandagopal P., Steven A.N., Chan L.W., Rahmat Z., Jamaluddin H. and Mohd Noh N.I. (2021) Bioactive metabolites produced by cyanobacteria for growth adaptation and their pharmacological properties. Biology 10, 1061 10.3390/biology10101061 34681158 PMC8533319

[7] Abrudan M.I., Smakman F., Grimbergen A.J., Westhoff S., Miller E.L., van Wezel G.P. et al. (2015) Socially mediated induction and suppression of antibiosis during bacterial coexistence. Proc. Natl. Acad. Sci. U.S.A. 112, 11054–11059 10.1073/pnas.1504076112 26216986 PMC4568218

[8] Aminov R.I. (2009) The role of antibiotics and antibiotic resistance in nature. Environ. Microbiol. 11, 2970–2988 10.1111/j.1462-2920.2009.01972.x 19601960

[9] Ratcliff W.C. and Denison R.F. (2011) Alternative actions for antibiotics. Science 332, 547–548 10.1126/science.1205970 21527704

[10] Davies J., Spiegelman G.B. and Yim G. (2006) The world of subinhibitory antibiotic concentrations. Curr. Opin. Microbiol. 9, 445–453 10.1016/j.mib.2006.08.006 16942902

[11] Yim G., Wang H.H. and Davies J. (2006) The truth about antibiotics. Int. J. Med. Microbiol. 296, 163–170 10.1016/j.ijmm.2006.01.039 16503195

[12] Linares J.F., Gustafsson I., Baquero F. and Martinez J.L. (2006) Antibiotics as intermicrobial signaling agents instead of weapons. Proc. Natl. Acad. Sci. U.S.A. 103, 19484–19489 10.1073/pnas.0608949103 17148599 PMC1682013

[13] Chevrette M.G., Gutiérrez-García K., Selem-Mojica N., Aguilar-Martínez C., Yañez-Olvera A., Ramos-Aboites H.E. et al. (2020) Evolutionary dynamics of natural product biosynthesis in bacteria. Nat. Prod. Rep. 37, 566–599 10.1039/C9NP00048H 31822877

[14] Kolter R. and van Wezel G.P. (2016) Goodbye to brute force in antibiotic discovery? Nat. Microbiol. 1, 15020 10.1038/nmicrobiol.2015.20 27571977

[15] Medema M.H. and van Wezel G.P. (2025) New solutions for antibiotic discovery: prioritizing microbial biosynthetic space using ecology and machine learning. PLoS Biol. 23, e3003058 10.1371/journal.pbio.3003058 40019875 PMC11878895

[16] Smanski M.J., Schlatter D.C. and Kinkel L.L. (2016) Leveraging ecological theory to guide natural product discovery. J. Ind. Microbiol. Biotechnol. 43, 115–128 10.1007/s10295-015-1683-9 26434742

[17] Rigali S., Anderssen S., Naômé A. and van Wezel G.P. (2018) Cracking the regulatory code of biosynthetic gene clusters as a strategy for natural product discovery. Biochem. Pharmacol. 153, 24–34 10.1016/j.bcp.2018.01.007 29309762

[18] Troxell B. and Hassan H.M. (2013) Transcriptional regulation by Ferric Uptake Regulator (Fur) in pathogenic bacteria. Front. Cell. Infect. Microbiol. 3, 59 10.3389/fcimb.2013.00059 24106689 PMC3788343

[19] Lee J.W. and Helmann J.D. (2007) Functional specialization within the Fur family of metalloregulators. Biometals 20, 485–499 10.1007/s10534-006-9070-7 17216355

[20] Lee N., Kim W., Chung J., Lee Y., Cho S., Jang K.S. et al. (2020) Iron competition triggers antibiotic biosynthesis in *Streptomyces coelicolor* during coculture with *Myxococcus xanthus*. ISME J. 14, 1111–1124 10.1038/s41396-020-0594-6 31992858 PMC7174319

[21] Dubey M., Meena M., Aamir M., Zehra A. and Upadhyay R. (2019) Regulation and role of metal ions in secondary metabolite production by microorganisms. New Future Dev. Microb. Biotechnol. Bioeng. 259–277 10.1016/B978-0-444-63504-4.00019-0

[22] Nguyen A.T., Jones J.W., Ruge M.A., Kane M.A. and Oglesby-Sherrouse A.G. (2015) Iron depletion enhances production of antimicrobials by *Pseudomonas aeruginosa*. J. Bacteriol. 197, 2265–2275 10.1128/JB.00072-15 25917911 PMC4524187

[23] Martinet L., Naômé A., Deflandre B., Maciejewska M., Tellatin D., Tenconi E. et al. (2019) A single biosynthetic gene cluster is responsible for the production of bagremycin antibiotics and ferroverdin iron chelators. mBio 10, e01230–19 10.1128/mBio.01230-19 31409675 PMC6692506

[24] Bignell D.R.D., Fyans J.K. and Cheng Z. (2014) Phytotoxins produced by plant pathogenic *Streptomyces* species. J. Appl. Microbiol. 116, 223–235 10.1111/jam.12369 24131731

[25] King R.R., Lawrence C.H. and Gray J.A. (2001) Herbicidal properties of the thaxtomin group of phytotoxins. J. Agric. Food Chem. 49, 2298–2301 10.1021/jf0012998 11368592

[26] Bischoff V., Cookson S.J., Wu S. and Scheible W.R. (2009) Thaxtomin A affects CESA-complex density, expression of cell wall genes, cell wall composition, and causes ectopic lignification in *Arabidopsis thaliana* seedlings. J. Exp. Bot. 60, 955–965 10.1093/jxb/ern344 19269997 PMC2652064

[27] Scheible W.R., Fry B., Kochevenko A., Schindelasch D., Zimmerli L., Somerville S. et al. (2003) An Arabidopsis mutant resistant to thaxtomin A, a cellulose synthesis inhibitor from *Streptomyces* species. Plant Cell. 15, 1781–1794 10.1105/tpc.013342 12897252 PMC167169

[28] Johnson E.G., Joshi M.V., Gibson D.M. and Loria R. (2007) Cello-oligosaccharides released from host plants induce pathogenicity in scab-causing *Streptomyces* species. Physiol. Mol. Plant Path. 71, 18–25 10.1016/j.pmpp.2007.09.003

[29] Deflandre B., Stulanovic N., Planckaert S., Anderssen S., Bonometti B., Karim L. et al. (2022) The virulome of *Streptomyces scabiei* in response to cello-oligosaccharide elicitors. Microb. Genom. 8, 000760 10.1099/mgen.0.000760 35040428 PMC8914351

[30] Jourdan S., Francis I.M., Kim M.J., Salazar J.J.C., Planckaert S., Frère J.M. et al. (2016) The CebE/MsiK transporter is a doorway to the cello-oligosaccharide-mediated induction of *Streptomyces scabies* pathogenicity. Sci. Rep. 6, 27144 10.1038/srep27144 27250236 PMC4890002

[31] Francis I.M., Jourdan S., Fanara S., Loria R. and Rigali S. (2015) The cellobiose sensor CebR is the gatekeeper of *Streptomyces scabies* pathogenicity. mBio 6, e02018 10.1128/mBio.02018-14 25714708 PMC4358012

[32] Kerff F., Jourdan S., Francis I.M., Deflandre B., Ribeiro Monteiro S., Stulanovic N. et al. (2023) Common scab disease: structural basis of elicitor recognition in pathogenic *Streptomyces* species. Microbiol. Spectr. 11, e01975–23 10.1128/spectrum.01975-23 37791952 PMC10714786

[33] Guan D., Grau B.L., Clark C.A., Taylor C.M., Loria R. and Pettis G.S. (2012) Evidence that thaxtomin C is a pathogenicity determinant of *Streptomyces ipomoeae*, the causative agent of *Streptomyces* soil rot disease of sweet potato. Mol. Plant Microbe. Interact. 25, 393–401 10.1094/MPMI-03-11-0073 22088193

[34] Liu J., Wang Y., He H., Dong S., Tang L., Yang E. et al. (2023) The leucine-responsive regulatory protein SCAB_Lrp modulates thaxtomin biosynthesis, pathogenicity, and morphological development in *Streptomyces scabies*. Mol. Plant Pathol. 24, 167–178 10.1111/mpp.13285 36478143 PMC9831280

[35] He H., Tang L., Song M., Chen H., Zou Y., Li X. et al. (2024) Lrp family regulator SCAB_Lrp2 responds to the precursor tryptophan and represses the thaxtomin biosynthesis in *Streptomyces scabies*. Mol. Plant Pathol. 25, e70036 10.1111/mpp.70036 39617959 PMC11609053

[36] Barry S.M., Kers J.A., Johnson E.G., Song L., Aston P.R., Patel B. et al. (2012) Cytochrome P450–catalyzed ​​​​​l-tryptophan nitration in thaxtomin phytotoxin biosynthesis. Nat. Chem. Biol. 8, 814–816 10.1038/nchembio.1048 22941045 PMC3522571

[37] Lauzier A., Goyer C., Ruest L., Brzezinski R., Crawford D.L. and Beaulieu C. (2002) Effect of amino acids on thaxtomin A biosynthesis by *Streptomyces scabies*. Can. J. Microbiol. 48, 359–364 10.1139/w02-031 12030709

[38] Sakuda S., Isogai A., Matsumoto S., Suzuki A. and Koseki K. (1986) The structure of allosamidin, a novel insect chitinase inhibitor, produced by *Streptomyces* Sp. Tetrahedron Lett. 27, 2475–2478 10.1016/S0040-4039(00)84560-8

[39] Suzuki S., Nakanishi E., Ohira T., Kawachi R., Nagasawa H. and Sakuda S. (2006) Chitinase inhibitor allosamidin is a signal molecule for chitinase production in its producing *Streptomyces* I. Analysis of the chitinase whose production is promoted by allosamidin and growth accelerating activity of allosamidin. J. Antibiot. (Tokyo) 59, 402–409 10.1038/ja.2006.57 17025016

[40] Suzuki S., Nakanishi E., Ohira T., Kawachi R., Ohnishi Y., Horinouchi S. et al. (2006) Chitinase inhibitor allosamidin is a signal molecule for chitinase production in its producing *Streptomyces* II. Mechanism for regulation of chitinase production by allosamidin through a two-component regulatory system. J. Antibiot. (Tokyo) 59, 410–417 10.1038/ja.2006.58 17025017

[41] Maan H., Itkin M., Malitsky S., Friedman J. and Kolodkin-Gal I. (2022) Resolving the conflict between antibiotic production and rapid growth by recognition of peptidoglycan of susceptible competitors. Nat. Commun. 13, 431 10.1038/s41467-021-27904-2 35058430 PMC8776889

[42] Rigali S., Titgemeyer F., Barends S., Mulder S., Thomae A.W., Hopwood D.A. et al. (2008) Feast or famine: the global regulator DasR links nutrient stress to antibiotic production by Streptomyces. EMBO Rep. 9, 670–675 10.1038/embor.2008.83 18511939 PMC2475330

[43] Świątek M.A., Urem M., Tenconi E., Rigali S. and van Wezel G.P. (2012) Engineering of *N*-acetylglucosamine metabolism for improved antibiotic production in *Streptomyces coelicolor* A3(2) and an unsuspected role of NagA in glucosamine metabolism. Bioengineered 3, 280–285 10.4161/bioe.21371 22892576 PMC3477696

[44] Świątek M.A., Tenconi E., Rigali S. and van Wezel G.P. (2012) Functional analysis of the *N*-acetylglucosamine metabolic genes of *Streptomyces coelicolor* and role in control of development and antibiotic production. J. Bacteriol. 194, 1136–1144 10.1128/JB.06370-11 22194457 PMC3294797

[45] Westhoff S., Kloosterman A.M., van Hoesel S.F.A., van Wezel G.P. and Rozen D.E. (2021) Competition sensing changes antibiotic production in *Streptomyces*. mBio 12, e02729–20 10.1128/mBio.02729-20 33563841 PMC7885098

[46] Okada B.K. and Seyedsayamdost M.R. (2017) Antibiotic dialogues: induction of silent biosynthetic gene clusters by exogenous small molecules. FEMS Microbiol. Rev. 41, 19–33 10.1093/femsre/fuw035 27576366 PMC5233716

[47] Tenconi E., Traxler M.F., Hoebreck C., van Wezel G.P. and Rigali S. (2018) Production of prodiginines is part of a programmed cell death process in *Streptomyces coelicolor*. Front. Microbiol. 9, 1742 10.3389/fmicb.2018.01742 30127771 PMC6087738

[48] Rastogi S., Marchal E., Uddin I., Groves B., Colpitts J., McFarland S.A. et al. (2013) Synthetic prodigiosenes and the influence of C-ring substitution on DNA cleavage, transmembrane chloride transport and basicity. Org. Biomol. Chem. 11, 3834–3845 10.1039/c3ob40477c 23640568

[49] Suryawanshi R.K., Patil C.D., Koli S.H., Hallsworth J.E. and Patil S.V. (2017) Antimicrobial activity of prodigiosin is attributable to plasma-membrane damage. Nat. Prod. Res. 31, 572–577 10.1080/14786419.2016.1195380 27353356

[50] Tenconi E. and Rigali S. (2018) Self-resistance mechanisms to DNA-damaging antitumor antibiotics in actinobacteria. Curr. Opin. Microbiol. 45, 100–108 10.1016/j.mib.2018.03.003 29642052

[51] Miguélez E.M., Hardisson C. and Manzanal M.B. (1999) Hyphal death during colony development in *Streptomyces* antibioticus: morphological evidence for the existence of a process of cell deletion in a multicellular prokaryote. J. Cell Biol. 145, 515–525 10.1083/jcb.145.3.515 10225953 PMC2185084

[52] Méndez C., Brana A.F., Manzanal M.B. and Hardisson C. (1985) Role of substrate mycelium in colony development in *Streptomyces*. Can. J. Microbiol. 31, 446–450 10.1139/m85-083 3891055

[53] Tenconi E., Traxler M., Tellatin D., van Wezel G.P. and Rigali S. (2020) Prodiginines Postpone the onset of sporulation in *Streptomyces coelicolor*. Antibiotics (Basel) 9, E847 10.3390/antibiotics9120847 PMC7760128 33256178

[54] Saubenova M., Rapoport A., Venkatachalam M., Dufossé L., Yermekbay Z. and Oleinikova Y. (2024) Production of carotenoids by microorganisms. Fermentation 10, 502 10.3390/fermentation10100502

[55] Villa F., Wu Y.L., Zerboni A. and Cappitelli F. (2022) In living color: pigment-based microbial ecology at the mineral–air interface. Bioscience 72, 1156–1175 10.1093/biosci/biac091 36451971 PMC9699719

[56] Lim S., Jung J.H., Blanchard L. and de Groot A. (2019) Conservation and diversity of radiation and oxidative stress resistance mechanisms in *Deinococcus* species. FEMS Microbiol. Rev. 43, 19–52 10.1093/femsre/fuy037 30339218 PMC6300522

[57] Muñoz-Torres P., Cárdenas-Ninasivincha S. and Aguilar Y. (2024) Exploring the agricultural applications of microbial melanin. Microorganisms 12, 1352 10.3390/microorganisms12071352 39065119 PMC11278939

[58] Tran-Ly A.N., Reyes C., Schwarze F.W.M.R. and Ribera J. (2020) Microbial production of melanin and its various applications. World J. Microbiol. Biotechnol. 36, 170 10.1007/s11274-020-02941-z 33043393 PMC7548279

[59] Konzen M., De Marco D., Cordova C.A.S., Vieira T.O., Antônio R.V. and Creczynski-Pasa T.B. (2006) Antioxidant properties of violacein: possible relation on its biological function. Bioorg. Med. Chem. 14, 8307–8313 10.1016/j.bmc.2006.09.013 17011197

[60] Behera A.K., Parida S., Mandal A.K., Patra S. and Jena M. (2025) Cyanobacterial scytonemin, a potential photoprotective natural pigment: biomedical, industrial and environmental applications. Arch. Microbiol. 207, 265 10.1007/s00203-025-04462-5 40956326

[61] Ribeiro Monteiro S., Kerdel Y., Gathot J. and Rigali S. (2025) The transcriptional architecture of bacterial biosynthetic gene clusters. J. Nat. Prod. 10.1021/acs.jnatprod.5c00529 40569150

[62] Urem M., Świątek-Połatyńska M.A., Rigali S. and van Wezel G.P. (2016) Intertwining nutrient-sensory networks and the control of antibiotic production in *Streptomyces*. Mol. Microbiol. 102, 183–195 10.1111/mmi.13464 27425419

[63] Ribeiro Monteiro S. and Rigali S. (2025) Enhanced prediction of expression control in bacterial biosynthetic gene clusters via genomic and functional data integration. Microb. Genom. 11, 001512 10.1099/mgen.0.001512 41091546 PMC12527672

